# cGMP-grade human iPSC-derived retinal photoreceptor precursor cells rescue cone photoreceptor damage in non-human primates

**DOI:** 10.1186/s13287-021-02539-8

**Published:** 2021-08-19

**Authors:** Swathi Lingam, Zengping Liu, Binxia Yang, Wendy Wong, Bhav Harshad Parikh, Jun Yi Ong, Debbie Goh, Daniel Soo Lin Wong, Queenie Shu Woon Tan, Gavin S. W. Tan, Graham E. Holder, Kakkad Regha, Veluchamy Amutha Barathi, Walter Hunziker, Gopal Lingam, Xianmin Zeng, Xinyi Su

**Affiliations:** 1 Institute of Molecular and Cell Biology (IMCB), Agency for Science, Technology and Research (A*STAR), Singapore, 138673 Singapore; 2 Department of Ophthalmology, Yong Loo Lin School of Medicine, National University of Singapore, Singapore, 117597 Singapore; 3 Singapore Eye Research Institute (SERI), Singapore, 169856 Singapore; 4 Department of Ophthalmology, National University Hospital, Singapore, 119074 Singapore; 5 Academic Clinical Program in Ophthalmology, Duke-NUS Medical School, Singapore, 169857 Singapore; 6 Department of Physiology, Yong Loo Lin School of Medicine, National University of Singapore, Singapore, 117593 Singapore; 7 RxCell Inc, Novato, CA 94949 USA; 8 UCL Institute of Ophthalmology, London, WC1E 6BT UK

**Keywords:** Cell therapy, Induced pluripotent stem cells, Non-human primates, Photoreceptor precursors

## Abstract

**Background:**

Retinal regenerative therapies hold great promise for the treatment of inherited retinal degenerations (IRDs). Studies in preclinical lower mammal models of IRDs have suggested visual improvement following retinal photoreceptor precursors transplantation, but there is limited evidence on the ability of these transplants to rescue retinal damage in higher mammals. The purpose of this study was to evaluate the therapeutic potential of photoreceptor precursors derived from clinically compliant induced pluripotent stem cells (iPSCs).

**Methods:**

Photoreceptor precursors were sub-retinally transplanted into non-human primates (*Macaca fascicularis*). The cells were transplanted both in naïve and cobalt chloride-induced retinal degeneration models who had been receiving systemic immunosuppression for one week prior to the procedure. Optical coherence tomography, fundus autofluorescence imaging, electroretinography, ex vivo histology and immunofluorescence staining were used to evaluate retinal structure, function and survival of transplanted cells.

**Results:**

There were no adverse effects of iPSC-derived photoreceptor precursors on retinal structure or function in naïve NHP models, indicating good biocompatibility. In addition, photoreceptor precursors injected into cobalt chloride-induced retinal degeneration NHP models demonstrated an ability both to survive and to mature into cone photoreceptors at 3 months post-transplant. Optical coherence tomography showed restoration of retinal ellipsoid zone post-transplantation.

**Conclusions:**

These findings demonstrate the safety and therapeutic potential of clinically compliant iPSC-derived photoreceptor precursors as a cell replacement source for future clinical trials.

**Supplementary Information:**

The online version contains supplementary material available at 10.1186/s13287-021-02539-8.

## Background

The global incidence of inherited retinal diseases (IRDs) is approximately 1 in 2000 [1]. These disorders are amongst some of the leading causes of blindness worldwide [2, 3]. IRDs are a group of genetically and clinically heterogeneous diseases with progressive retinal damage leading to vision loss [4]. Retinitis pigmentosa (RP, rod-cone dystrophy) is the most common with approximately 1.5 million people affected worldwide [5].

The introduction of gene therapy, such as Voretigene Neparvovec-rzyl-Luxturna™ for the treatment of biallelic Retinal Pigment Epithelium 65 (RPE65) associated Leber Congenital Amaurosis (LCA), has been a significant development [6–9]. However, the sheer extent of genetic heterogeneity, with more than 260 genes implicated in IRDs [3], limits the widespread application of gene therapy for all IRDs. Moreover, gene therapy will have limited efficacy in clinical cases of advanced retinal degeneration in which significant photoreceptor cell death has already occurred [10].

With the advent of induced pluripotent stem cell (iPSC) and embryonic stem cell (ESC) technology, regenerative stem cell therapy has the potential to be an alternative treatment for end-stage retinal degeneration, independent of the underlying genetic defect. ESC- and iPSC-derived retinal progenitor cells (RPCs) or photoreceptor precursors can differentiate into photoreceptors [11], making them an attractive cell therapy resource [12–14]. A number of studies in rodent models of photoreceptor degeneration have demonstrated that photoreceptors precursors injected sub-retinally are capable of differentiating into photoreceptors, albeit with limited survival and integration with host tissue [10, 15–17]. More recent efforts, focused on transplanting retinal sheets consisting of both neural retina and retinal pigment epithelium (RPE) derived from iPSC/ESC into both rodent and non-human primate (NHP) models [18–21], have suboptimal outcomes due to tissue disorganization (inappropriate photoreceptor orientation) and poor integration with host bipolar cells [19, 21].

The use of foetal and embryonic stem cells is restricted by limited access to new donor tissue and potential ethical concerns. Thus, allogenic iPSC RPC presents an attractive alternative cell resource. However, there is a potential risk of genetic instability introduced during re-programming [10] and it is imperative to develop Current Good Manufacturing Practice (cGMP) standards to ensure the generation of clinically compliant and safe iPSC.

This study evaluates the safety and efficacy of cGMP-iPSC-derived photoreceptor precursors injected sub-retinally into naïve NHP models, and their ability to promote endogenous photoreceptor structural recovery in cobalt chloride (CoCl_2_)-induced NHP models of retinal degeneration [21]. The photoreceptor precursors were injected as a cell suspension as they were produced from a two-dimensional (2D) cell culture system. The methodology used, which incorporates in vivo optical coherence tomography (OCT), examines biocompatibility with the naïve NHP eye; ability to promote ellipsoid zone recovery following CoCl_2_ damage; and ability of photoreceptor precursors to survive in the outer nuclear layer (ONL) and differentiate into cone photoreceptors.

## Methods

### RPC maintenance and maturation into photoreceptor precursors

The cGMP-grade iPSC line used in this study was obtained from RxCell Science as described previously [22, 23]. This is the first USFDA-approved iPSC line developed under cGMP-complaint conditions including tissue sourcing, manufacturing, testing and storage. The line was differentiated into RPC for 8 weeks at RxCell under cGMP conditions, cryopreserved and shipped to Singapore. Subsequent maturation for another 4 weeks (12 weeks old in total) before transplantation was performed in Singapore under non-cGMP conditions. These RPCs were plated at a cell density of 1 to 2 million in 6-well plates pre-coated with CTG-Retinal Coating Substrate (XR-CTG-101-SUBS, RxCell Science, US) in cGMP-grade Retinal Differentiation Medium (RD-001-M100, RxCell Science, US) for 12 weeks to obtain photoreceptor precursors for transplantation.

### Characterization of RPCs and photoreceptor precursors by immunocytochemistry

Cells were cultured in 12-well microscopy chamber slides (#81201, Ibidi, Munich, Germany) and stained for stem cell, retinal progenitor and cone/rod photoreceptor markers (Additional file 1: Table S1). The cells were fixed with 4% paraformaldehyde (PFA) for 10 min at room temperature, blocked in 1% bovine serum albumin (BSA) and incubated with primary antibodies (diluted in 1% BSA) overnight at 4 °C. The cells were counterstained with Alexa Fluor 488, Alexa Fluor 568, Alexa Fluor 647 and Hoechst 33342 at room temperature for 30 min and mounted in ProLong™ Gold Anti-fade Mountant (Thermo Fisher Scientific, P36930, Waltham, Massachusetts, USA). Fluorescence micrographs were collected using a Zeiss LSM 800 confocal microscope (Carl Zeiss GmBH, Jena, Germany). The expression of each marker was quantified from at least five micrographs using Fiji version 1.46r [29].

### Lentivirus packaging

Lentivirus pCDH-GFP was packaged in H293T cells using TransITLT1 transfection reagent (Mirus Bio, MIR 2305, Madison, Wisconsin, USA). Briefly, pCDH-GFP plasmid was co-transfected into H293T cells together with tat, rev, gag/pol and vsv-g packaging plasmids. Virus particles were harvested at 48 h and 72 h post-transfection, filtered with a 0.45 mm filter (17,574-K, Sartorius, Göttingen, Germany) and concentrated by ultracentrifugation at 23,000 rpm for 90 min. The virus was stored in 100 µL aliquots at − 80 °C.

### GFP labelling of photoreceptor precursors and dissociation for transplantation

Lentivirus infection was performed one week prior to transplantation. 5 × 10^7^/mL (Multiplicity of infection = 5) of pCDH-GFP virus was mixed with 4 µg of polybrene (Sigma-Aldrich, TR-1003-G, St. Louis, Missouri, USA) in 1 mL of culture medium and added to the cells for 6 h, after which it was replaced by fresh culture medium. The photoreceptor precursors were dissociated into single cells on the day of transplantation using TrypLE™ Express Enzyme (Thermo Fisher Scientific, 12604013, Waltham, Massachusetts, USA) as previously described [15]. Infection efficiency was analysed by flow cytometry (BD FACS LSR II, BD Bioscience, San Jose, California, USA), and culture plates were also imaged using a Zeiss Axio Vert A1 inverted microscope (Carl Zeiss GmBH, Jena, Germany) to detect GFP expression.

### Animal studies

A total of six *Macaca fascicularis*, aged 3 to 5 years old and weighing 3 to 4.5 kg were sourced from SingHealth Experimental Medicine Centre, Singapore (IACUC numbers: 2015/SHS/1092, 2015/SHS/1044). All animal experiments were approved by the Institutional Animal Care and Use Committee (IACUC) of SingHealth (Singapore) (IRB number: 2019-009), performed in an American Association for Accreditation of Laboratory Animal Care (AAALAC) approved facility, and according to the Statement from the Association for Research in Vision and Ophthalmology (ARVO) for the Use of Animals in Ophthalmic and Vision Research.

### Cobalt chloride-induced NHP retinal degeneration model

Cobalt chloride hexahydrate (CoCl_2_·6H_2_O, molecular weight of 237.93, Nacalai tesque, Kyoto, Japan) was dissolved in 0.9% saline solution to obtain 0.3 mg/mL solution for induction of retinal injury [21]. Animals were sedated with ketamine (10 mg/kg, body weight (BW)) and atropine (0.05 mg/kg, BW) followed by induction of general anaesthesia (GA) with 2% Isoflurane and maintenance with 0.5–2% Isoflurane [24, 25]. A 25-gauge (G) 3 port-vitrectomy (infusion, chandelier endo-illumination, and working port) was performed with a Bausch & Lomb Stellaris® PC machine (Bausch & Lomb, Rochester, New York, USA). Local retinal detachment (1- to 2-disc diameter size) within macular region was created by manual sub-retinal injection of 40 to 50 µL of 0.3 mg/mL CoCl_2_ solution using an extendible 38 G sub-retinal injection cannula [26] (MedOne Surgical Inc., Sarasota, Florida, USA). Retinal and RPE damage was observed by OCT and Fundus Autofluorescence (FAF) imaging.

### Immunosuppression

All NHPs received systemic immunosuppression 7 days prior to photoreceptor precursor transplantation, and this was continued throughout the study period [25]. Sirolimus was administered orally with a loading dose of 2 mg, followed by a 1 mg daily dose. Doxycycline and minocycline (7.5 mg/kg, BW) were delivered orally, twice daily [24, 27].

### Transplantation of photoreceptor precursors into NHPs

In both naïve and CoCl_2_-induced diseased NHPs, a suspension of photoreceptor precursors in media was manually injected sub-retinally using an extendible 38 G injection cannula (MedOne Surgical Inc., Sarasota, Florida, USA)(Additional file 2). Seven eyes from 6 NHPs received photoreceptor precursors at two different dosages (40,000 to 60,000 cells, *n* = 3, for initial safety test in naïve NHPs; 100,000 to 300,000 cells, *n* = 4 for rescue in diseases NHPs). The retinal bleb was created by injection of 40 to 50 µL cell suspension medium with about 1- to 2-disc diameter size. Details are provided in Table 1.

**Table 1:** Summary of sub-retinal injection of cGMP-iPSC-derived photoreceptor precursors in both naïve and disease NHP models

Period of Cocl_2_ damage	No. of cells	GFP labelled	Period of rescue	Eyes
Naïve				
N.A	40,000–60,000	Non-GFP labelled	3 months	n = 3
Disease/rescue				
1 month	100,000–300,000	GFP labelled	1 month	n = 1
1 month	100,000–300,000	GFP labelled	3 months	n = 1
4 months	100,000–300,000	GFP labelled	3 months	n = 2

### In vivo follow up by ophthalmic imaging

Cross-sectional images of the central 30 degrees of the retina were acquired non-invasively using spectral domain-optical coherence tomography (SD-OCT, Spectralis® Heidelberg Engineering, Inc., Heidelberg, Germany). Real-time eye-tracking system and image registration capabilities on the Spectralis® were used to mitigate motion artefacts. Repeat OCT scans were performed at the same location over consecutive evaluations. Fundus auto-fluorescence (FAF) and infrared (IR) images were obtained using the same device.

### Qualitative SD-OCT analysis

Precise mapping of the area and extent of sub-retinal CoCl_2_ and photoreceptor precursor injection was achieved by extrapolating the information from surgical videos super-imposed onto the OCT images, using vessel markings as landmarks. Treated areas of the retina were analysed for structural changes. The ellipsoid zone (EZ), identified as the second hyper-reflective band on the OCT image, was used as a surrogate imaging biomarker of photoreceptor integrity. Disruption of the EZ was the indicator of the presence of photoreceptor damage, and restoration of the EZ is an indicator of photoreceptor recovery.

### Electroretinography

Global retinal function was assessed by full-field electroretinography (ERG) using an Espion system (Diagnosys LLC, Lowell, Massachusetts, USA) and Jet recording electrodes with protocols based upon those recommended for humans by the International Society for Clinical Electrophysiology of Vision (ISCEV) [28] but using a flash strength of 5 cd s m^−2^ for the photopic single flash responses.

### Histopathological examination

Animals were sacrificed under deep anaesthesia and perfused with 10% formalin at 3 months after photoreceptor precursor transplantation. The eyes were enucleated and fixed overnight. Full-thickness foveal samples (retina → sclera) were collected and embedded in paraffin. 10 µm sections were cut with a microtome (Leica RM2255, Wetzlar, Germany) and stained with hematoxylin and eosin (H&E).

### Immunofluorescence staining of NHP retinal sections

Paraffin-embedded sections were deparaffinized in xylene and rehydrated in ethanol. Following antigen retrieval at 95 °C for 20 min in citrate buffer pH 7.0 or tris-EDTA buffer pH 9.0 (depending on the antibody), the samples were blocked for 1 h in 10% donkey serum. Samples were incubated with primary antibodies overnight at 4 °C (Additional file 1: Table S1). The samples were counterstained with Alexa 488, Alexa 568 and Alexa 647, mounted in ProLong™ Gold Antifade Mountant (Thermo Fisher Scientific, P36930, Waltham, Massachusetts, USA) and imaged using a Zeiss LSM800 upright confocal microscope (Carl Zeiss GmBH, Jena, Germany). Z-stack images were collected every 0.56 μm, and the micrographs were analysed using Fiji version 1.46r [29].

## Results

### Characterization of photoreceptor precursors in vitro prior to transplantation

**Fig. 1:**
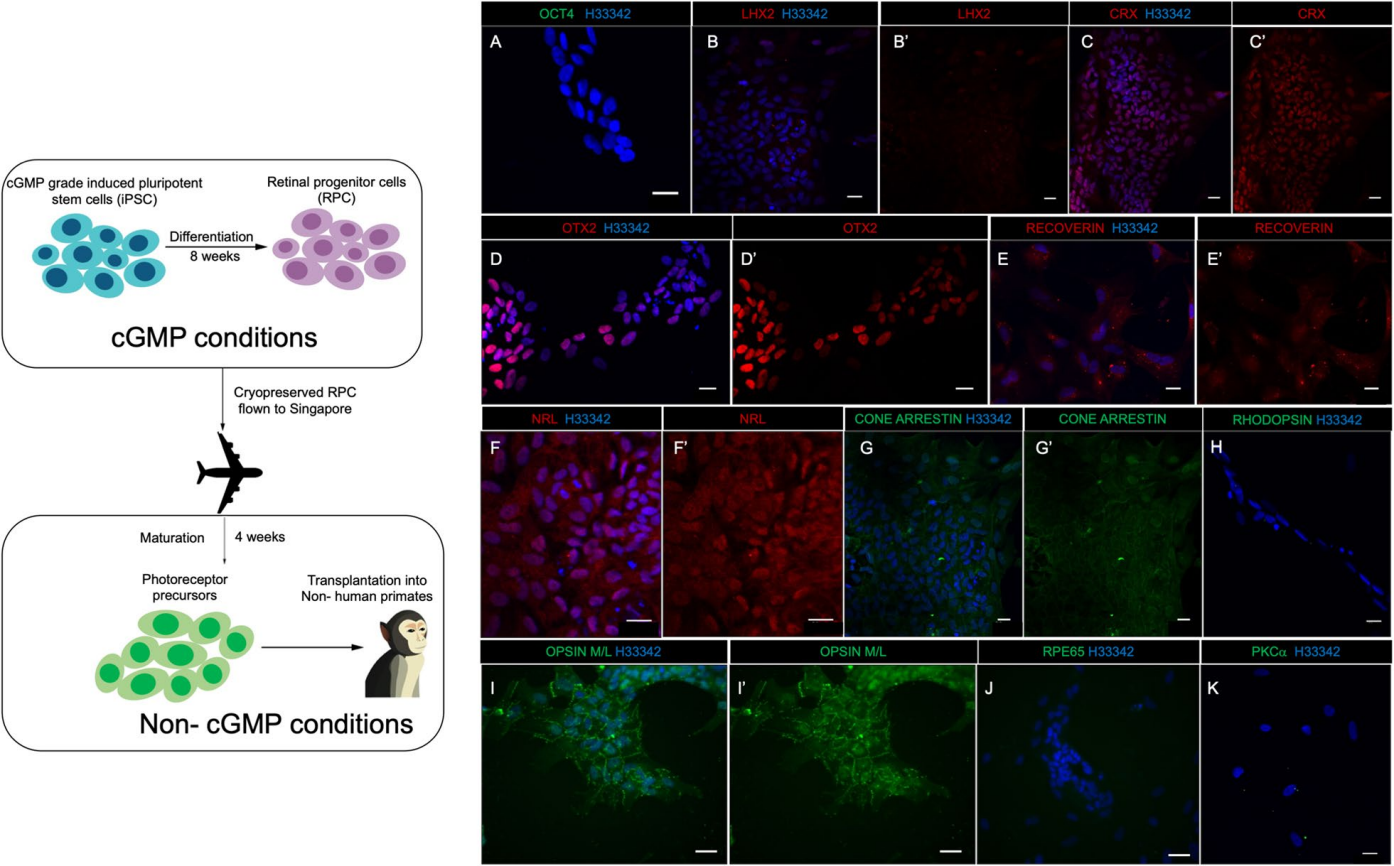
IF staining of photoreceptor precursors. The cells at 12 weeks old showed absence of pluripotency marker OCT4 (**A**) and only a minority of cells expressed the retinal progenitor marker LHX2 (**B**, **B’**). Majority of the cultures expressed pan-photoreceptor markers CRX (**C**, **C’**), OTX (**D**, **D’**), Recoverin (**E**, **E’**). In addition, presence of early rod-specific transcription factor NRL (**F**, **F’**) and cone-specific markers cone arrestin (**G**, **G’**) and opsin M/L (**I** and **I’**), but not the RPE cell marker RPE65 (**J**), nor the bipolar cell marker PKCα (**H**) is suggestive of commitment to the photoreceptor lineage. Of note, we did not detect the rod marker rhodopsin after 12 weeks of culture (**H**). Nuclei are labelled with Hoechst 33342 (H33342). Scale bar: 20 µm. The schematic on the left represents the cell resource development process

Cells were thawed and cultured for an additional 4 weeks prior to transplantation. The cells, thus 12 weeks old, showed the absence of pluripotency marker OCT4 (Fig. 1A) and only a minority of cells express the retinal progenitor marker LHX2 (Fig. 1B and B’). The majority of the cells express pan-photoreceptor markers CRX (37 ± 7% of total Hoechst 33342 stained cells) (Fig. 1C and C’), Recoverin (41 ± 3% of total Hoechst 33342 stained cells) (Fig. 1E and E’) and OTX2 (46.3 ± 13% of total Hoechst 33342 stained cells) (Fig. 1D and D’). These markers were previously used to characterize photoreceptor precursors derived from ESC and retinal organoids [30–32]. The expression levels of the pan-photoreceptor markers were comparable to previously published data for photoreceptor precursors derived from the same cGMP-grade iPSCs [15]. In addition, the presence of early rod-specific transcription factor NRL (45 ± 16% of total Hoechst  33342 stained cells) (Fig. 1F and F’) and the cone-specific markers cone-arrestin (43 ± 2.6% of total Hoechst 33342 stained cells) (Fig. 1G and G’) and Opsin M/L (46.65 ± 1.67% of total Hoechst 33342 stained cells) (Fig. 1I and I’) was observed, indicating that these cells at 12 weeks old were photoreceptor precursors. Neither the bipolar cell marker PKCɑ (Fig. 1K) nor the RPE marker RPE65 (Fig. 1J) was observed, confirming the commitment to the photoreceptor lineage. Of note, these photoreceptor precursors did not express late rod specific marker rhodopsin, suggesting that they had not achieved the level of maturity needed for rhodopsin expression (Fig. 1H).

### Tolerance of transplantation of photoreceptor precursors into naïve NHP eyes

**Fig. 2:**
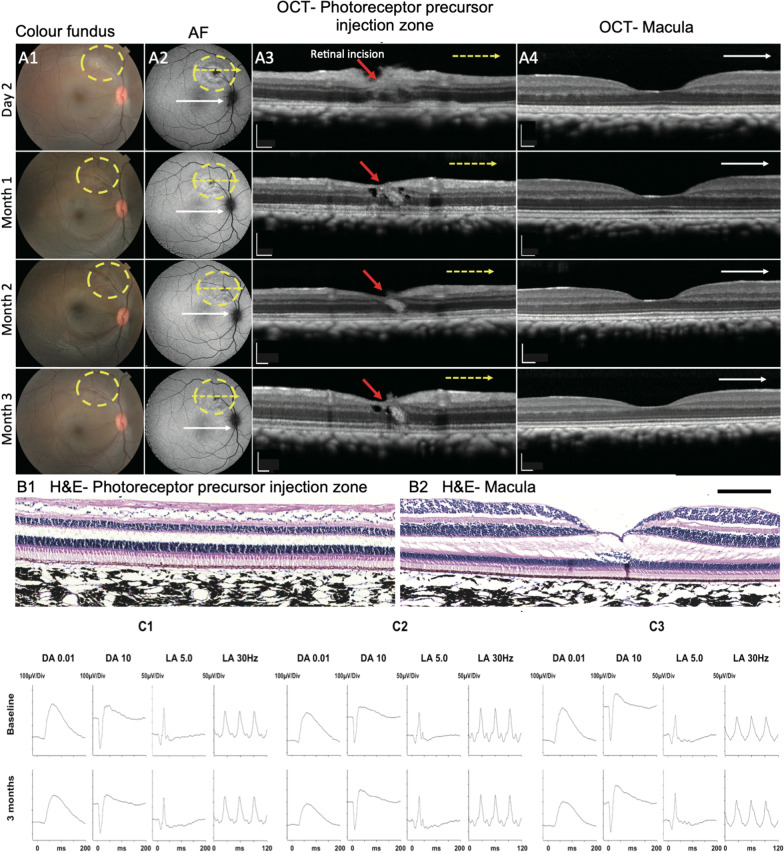
Safety of sub-retinal transplant photoreceptor precursors into naïve NHP eyes. **A** Photoreceptor precursors were transplanted subretinally in yellow circle zone (colour fundus, Column **A1**, and autofluorescence images, Column **A2**) in naïve NHP eyes and followed at 2 days, 1, 2 and 3 months post-transplantation. Limited changes were observed at transplantation zone on autofluorescence images and showed time-dependent recovery. OCT line scan images on transplantation zone (Column **A3**, indicated as yellow arrows in Column **A2**) showed interrupted ONL at retinal incision site (red arrows) at day 2 and recovered during follow up. Normal macula structures were shown on OCT scans (Column **A4**) as indicated the scanned position at white arrows in Column **A2**. **B** Histology images (H&E) on transplantation zone and macula both showed normal retinal structure. Scale bar, 200 μm in Column **A3** and **A4**, **B1** and **B2**. **C** Full-field electroretinography (ERG) was normal at 3-month follow-up in the eyes with transplanted photoreceptor precursors. **C1**, **C2** and **C3** each represent ERG recordings from 3 different NHPs. The terms DA and LA refer to dark-adapted and light-adapted. The numbers refer to flash strength in cd s m^−2^

Three naïve NHPs eyes received sub-retinal injections of 40,000 to 60,000 photoreceptor precursors for initial safety and biocompatibility evaluation (*n* = 3). Colour fundus and FAF images revealed a clear vitreous cavity with no overt signs of inflammation (Fig. 2A1 and A2). The initial disruption of the retinal layers at the injection site (Fig. 2A3) showed limited recovery at 3 months with residual scarring only at the original site of retinotomy, with normal surrounding retina. The structure of the macula was well maintained over the 3 months as shown by OCT imaging (Fig. 2A4) and histology (Fig. 2B2). Full-field ERG at 3 months post-transplantation showed no significant abnormality of retinal function in either cone or rod derived responses (Fig. 2C). All showed slightly lower DA 0.01 dim flash dark-adapted responses, but no definite clinically significant differences compared to the pre-treatment data.

### The effects of CoCl_2_ on the photoreceptors and retinal pigment epithelium in NHP eyes

**Fig. 3:**
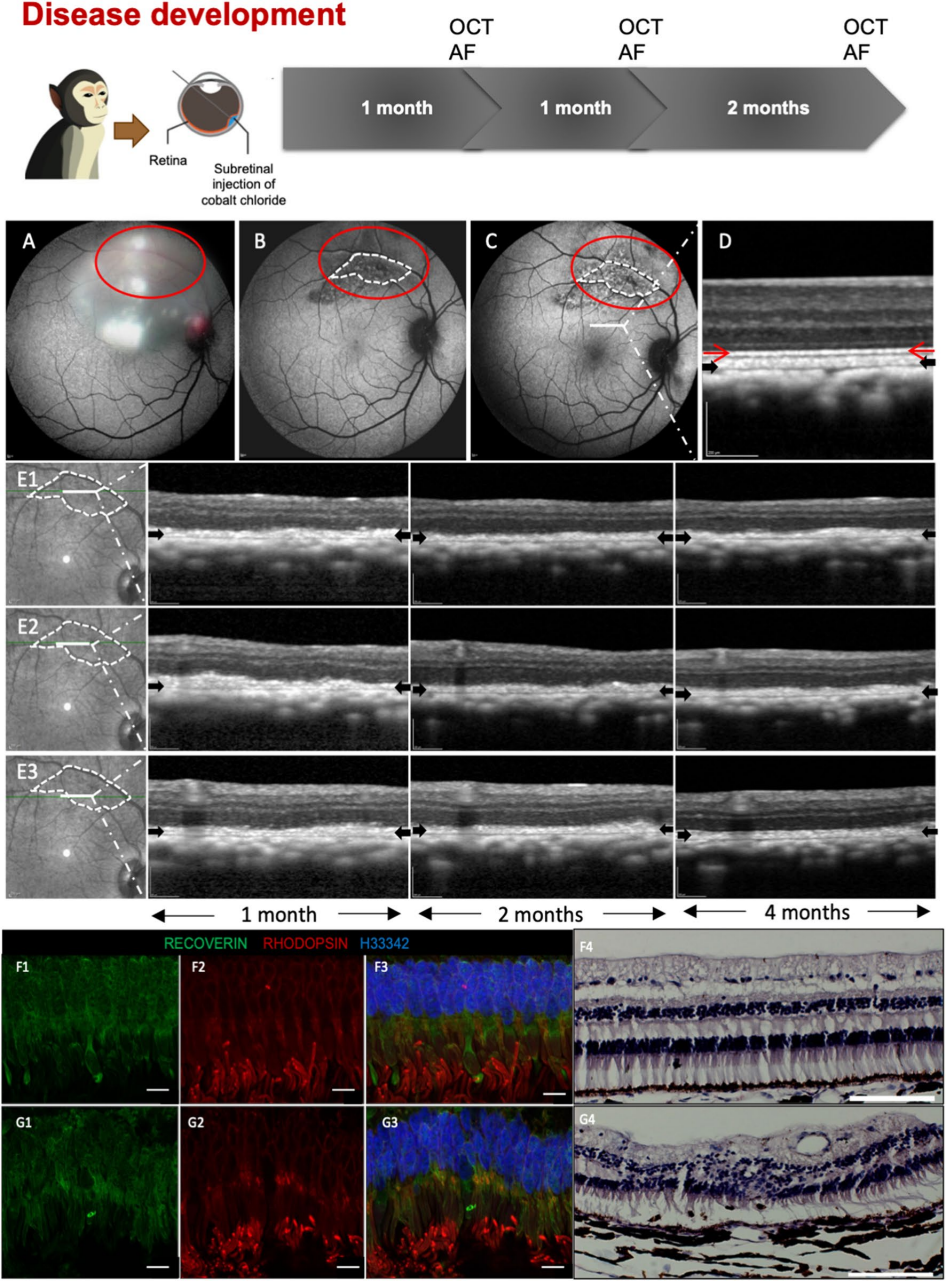
Establishment of CoCl_2_-induced retinal degeneration NHP model. **A** Intraoperative imaging of CoCl_2_ injection superimposed on fundus autofluorescence (FAF) image. **B** and** C** FAF images (1- and 4-month, respectively) showed persistently decreased autofluorescence signal intensities suggestive of RPE damage in the site of CoCl_2_ injected region within the red dotted lines. The area outlined in white was selected for analysis of morphological changes. **D** OCT scan showed normal retinal structure outside the CoCl_2_ injection zone. The red and black arrows indicate the intact ellipsoid zone and RPE/Bruch’s membrane complex, respectively. **E1** to **E3** tracked OCT line scans from different regions across the CoCl_2_ injected zone demonstrated persistent outer retinal alterations without recovery of the ellipsoid zone up to 4 months post-CoCl_2_ injection. Scale bars, 200 μm. **F1**–**F4** Immunofluorescence and H&E staining of retina in un-operated controls show ordered outer nuclear layer and intact photoreceptor outer segments. Scale bar: 10 μm. **G1**–**G4** Immunofluorescence and H&E staining of retina 1 month after CoCl_2_ damage shows disorganization of the outer nuclear layer and photoreceptor outer segments. Scale bar: 100 μm

In the NHP model of retinal degeneration established by sub-retinal injection of CoCl_2_ [33], mild to moderate ONL loss was observed on OCT imaging. High-resolution OCT scans revealed persistent outer retinal alterations and disruption of EZ for up to 4 months post-CoCl_2_ injection (Fig. 3E1 to E3). This was consistent with the histology data, which showed both ONL and inner nuclear layer (INL) damage compared to non-operated controls (Fig. 3F4 and G4). Immunofluorescence staining with the pan photoreceptor marker recoverin and the rod photoreceptor marker rhodopsin also showed disorganization of the outer nuclear later and photoreceptor outer segments 1 month after CoCl_2_ damage (Fig. 3G1-G3) compared with non-operated controls (Fig. 3F1-3). Corresponding FAF images showed persistent hyper-autofluorescence at the site of CoCl_2_ injection up to 4 months (Fig. 3B and C), in keeping with structural damage caused by CoCl_2_ treatment to both the photoreceptors and RPE.

### Effect of transplanted photoreceptor precursors on outer retinal layers in CoCl_2_ disease models

**Fig. 4:**
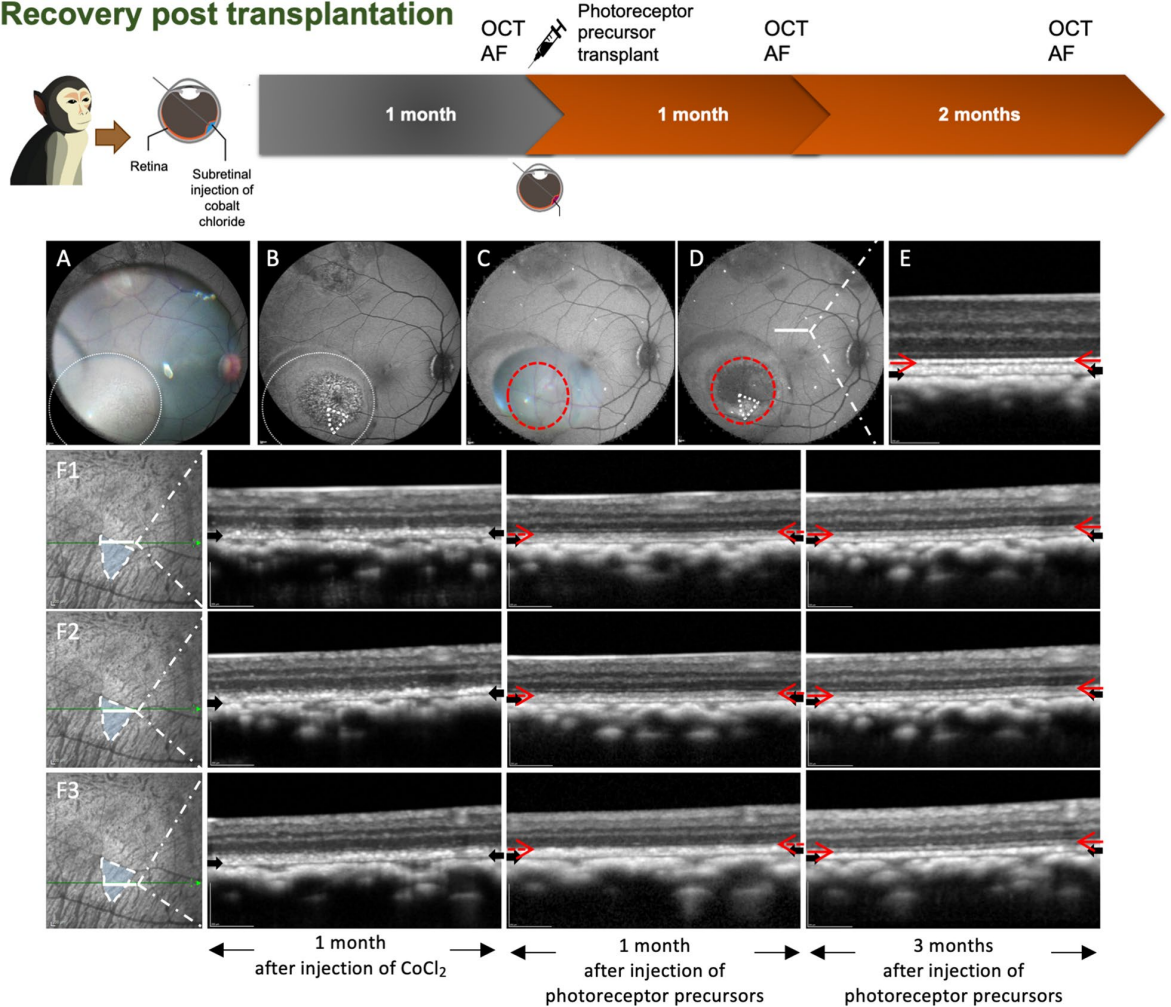
Retinal structure recovery in CoCl_2_ damaged zone by photoreceptor precursors injection. **A** Intraoperative imaging of CoCl_2_ injection superimposed on fundus autofluorescence (FAF) image. The site of CoCl_2_ was within the white dotted circle. **B** FAF imaging 1 month post-CoCl_2_ injection and immediately prior to photoreceptor precursors transplantation. **C** Intraoperative imaging of retinal photoreceptor precursors injection superimposed on the FAF image. The site of photoreceptor precursors was demarcated within the red dotted line. **D** FAF imaging was performed 4 months post-CoCl_2_ injection and 3 months post-photoreceptor precursors injection. The area outlined by the white dotted triangle was selected for analysis of morphological changes. The autofluorescence signal intensities in this zone reverted to normal suggesting recovery of RPE function. **E** OCT scan showed normal retinal structure outside the CoCl_2_ injected zone. The red and black arrows indicate the intact ellipsoid zone and RPE/Bruch’s membrane complex, respectively. **F1** to **F3** tracked OCT line scans of three different regions within the photoreceptor precursors injected zone. The ellipsoid zone was indistinct at 1 month post-CoCl_2_ injection but demonstrated progressive recovery with near-complete restoration at 3 months post-photoreceptor precursors injection

Photoreceptor precursors were injected sub-retinally into the damaged retinas of four eyes (100,000 to 300,000) to assess the ability of the transplanted cells to rescue retinal damage. In eyes rescued at 1 month post-CoCl_2_ damage, high-resolution, tracked SD-OCT line scans showed a reappearance of EZ signals 1 month post-injection, with continued improvement and near-complete restoration by 3 months post-injection (Fig. 4F1-3). In addition, at 3 months post-photoreceptor precursor injection, the hyper-auto fluorescence seen at 1 month post-CoCl_2_ injection (Fig. 4B) was reduced in intensity (Fig. 4C). This was consistent with OCT images showing continuity of the RPE layer in the regions rescued with photoreceptor precursor injection at 3 months (Fig. 4F).

### Maturation of transplanted photoreceptor precursors within the ONL of NHP retina

**Fig. 5:**
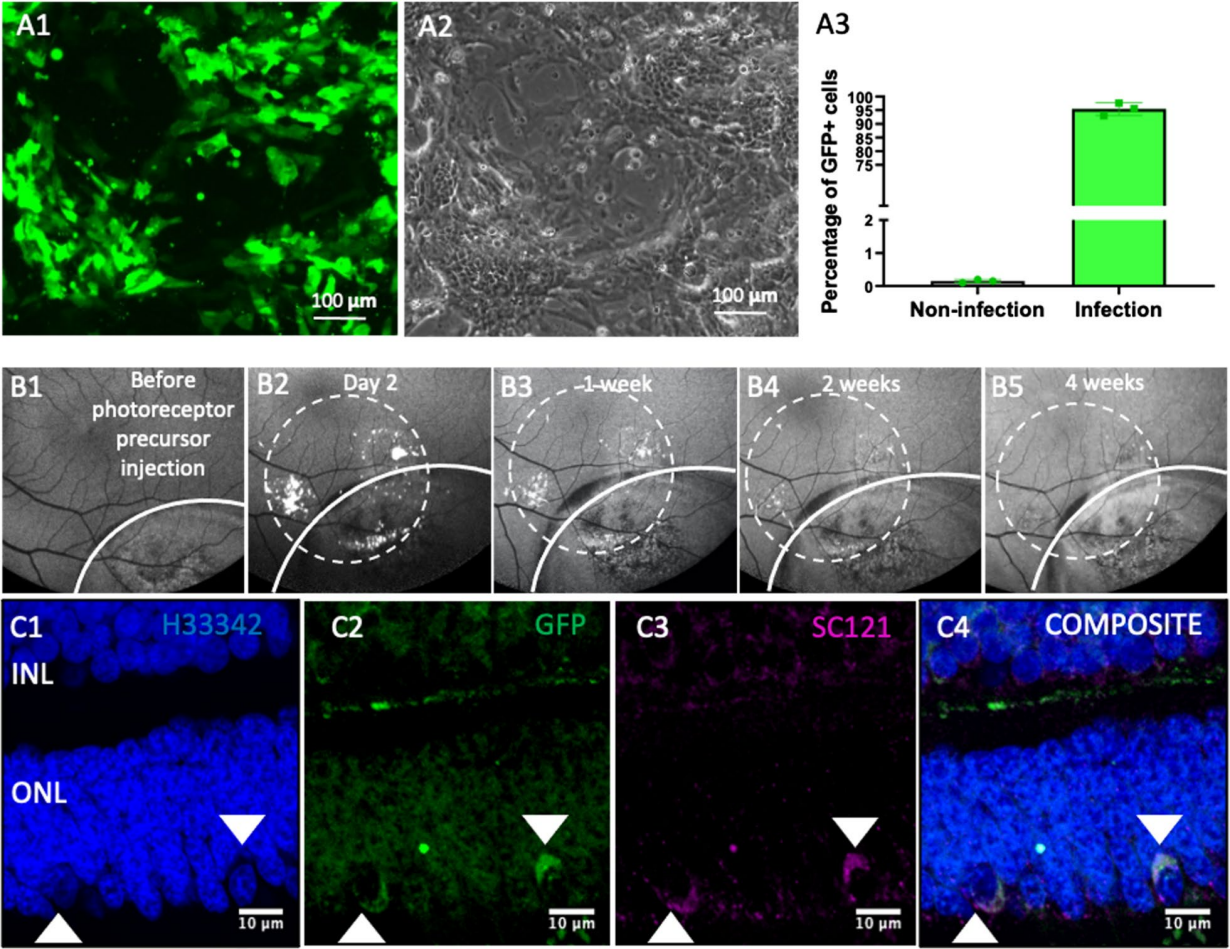
GFP-labelled photoreceptor precursors culture and in vivo tracking post-transplantation in NHP. **A1** Representative fluorescence micrograph for GFP labelling by pCDH-GFP lentivirus injection. **A2** Phase contrast image of the cells in **A1**. **A3** Quantification of GFP labelling efficiency (*n* = 3) by flow cytometry. 95% of the cells were labelled. **B1**–**B5** fundus autofluorescence images of in vivo follow up of transplanted photoreceptor precursors. The white curved lines indicated the region injected with CoCl_2_. The white dotted circles indicated the region injected with GFP-labelled photoreceptor precursors suspension. **C1** to **C4** Immunofluorescence staining of paraffin embedded tissues collected 12 weeks post-photoreceptor precursors transplant show GFP-positive (**C2**, white arrows) and SC121-positive (**C3**, white arrows) cells in the outer nuclear layer (ONL). The GFP- and SC121-positive cells co-localize (**C4**, white arrows), indicating that they are transplanted human photoreceptor precursors. Scale bars, 100 µm in **A1** and **A2**, 200 μm in **B1** to **B5**, 10 μm in **C1** to **C4**

Lentiviral transduction of the photoreceptor precursors was used to generate stable GFP expression to track transplanted cell survival in vivo and ex vivo. This was performed 1 week prior to transplantation with more than 90% efficiency as assessed by flow cytometry (Fig. 5A3, Additional file 1: figure S1). GFP-labelled photoreceptor precursors were transplanted sub-retinally into four eyes in CoCl_2_-induced diseased NHPs. Post-injection, the GFP signal was tracked by FAF imaging. GFP-positive cells were observed within the injection zone at 2 days post-surgery (Fig. 5B2), with gradual reduction by 1 to 2 weeks (Fig. 5B3 and B4) and were virtually undetectable by 4 weeks (Fig. 5B5). Live cell detection was likely limited by the relative low sensitivity of this method for low cell numbers. Long-term survival of GFP-labelled photoreceptor precursors was confirmed by immunofluorescence staining (Fig. 5C2) of retina sections at 3 months post-transplantation. GFP-positive cells were identified in the ONL and co-stained with a human cytoplasmic marker (SC121), confirming that these cells were of human origin (Fig. 5C).

**Fig. 6:**
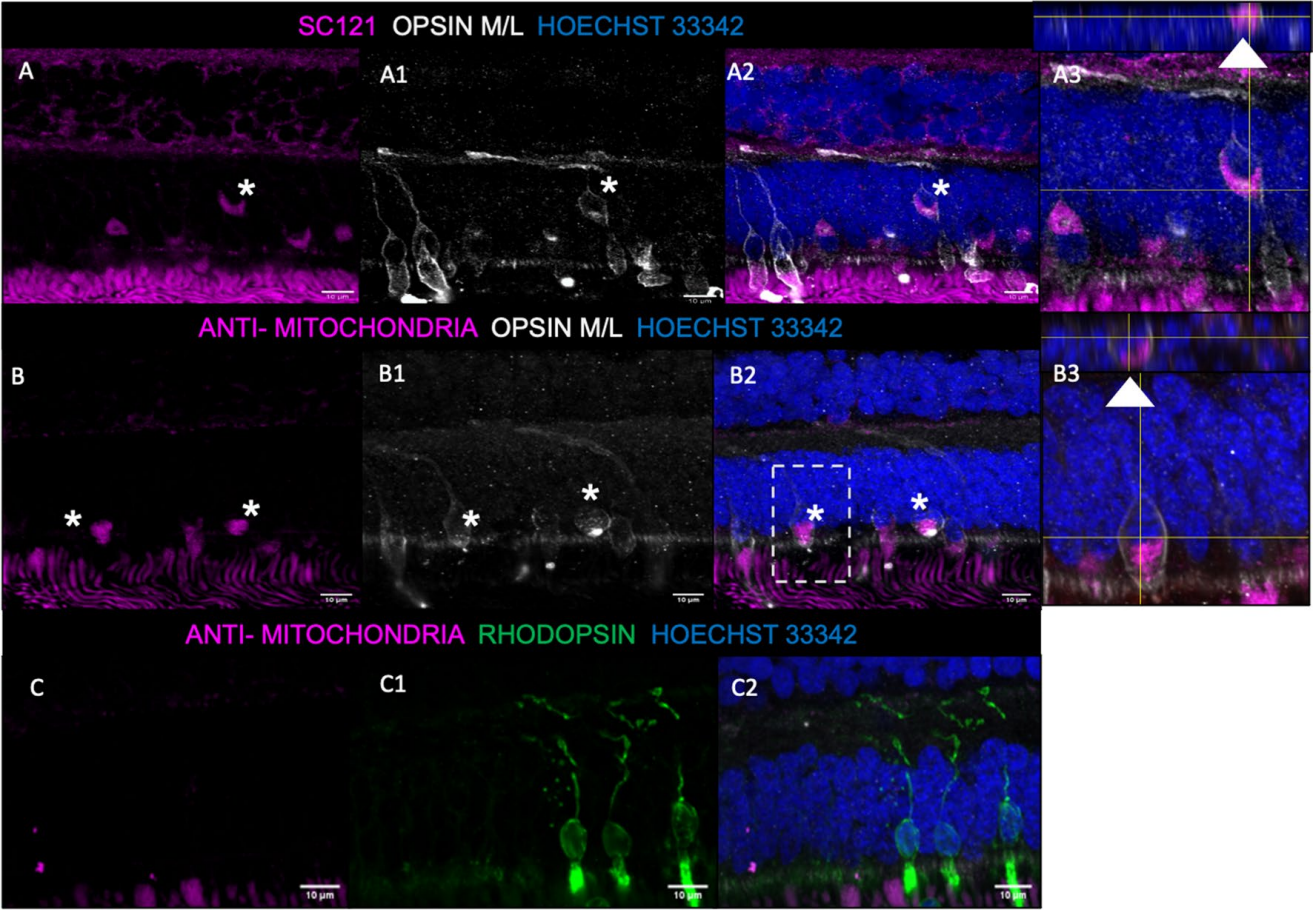
Survival and differentiation of transplanted photoreceptor precursors in the host NHP retina. **A**–**A3**, Anti-human SC121-positive cells appeared to survive in the outer nuclear layer (ONL) of the host NHP retina (**A** & **A2**, magenta). These cells co-localized with opsin M/L-positive staining (**A2**, grey, asterisk), suggestive that the transplanted photoreceptor precursors are able to differentiate into cones. Z-stack projection show the markers localized to a single cell (**A3**, white arrow). **B**–**B3**, Anti-human mitochondrial antibody (AMA)-positive photoreceptor precursors appeared to survive in the ONL of the host NHP retina (**B** & **B2**, magenta). They also co-localized with opsin M/L-positive cone photoreceptors (**B2**, grey, asterisk). Z-stack projection showed clear co-localization between AMA and opsin M/L around a nucleus (**B3**, white arrow). **C**–**C2**, AMA-positive photoreceptor precursors (**C** & **C2**, magenta) did not co-localize with rhodopsin-positive rod photoreceptors (**C2**, green). Nuclear staining with Hoechst 33342 (**A2**, **B2**, **C2**, blue) was used to identify the inner and outer nuclear layers. The cells not highlighted with an asterisk are most likely native NHP opsin M/L-positive cones. Scale bars: 10 μm

At 1 month post-transplantation, there was evidence of the precursors maturing into cone photoreceptors. Human-specific makers SC121 and anti-human mitochondria (AMA) were used to identify transplanted cells. SC121-positive (Fig. 6A) and AMA-positive (Fig. 6B) cells were identified in the ONL, and they co-localized with medium/long (M/L) opsin-positive staining, indicating maturation into cone photoreceptors (Fig. 6A1, B1 and C1). Human-derived cells were surrounded by SC121 and AMA negative cone photoreceptors, likely native NHP cones (Fig. 6A-A2 and B-B2). Transplanted photoreceptor precursor cells positive for AMA did not appear to co-localize with rhodopsin (Fig. 6C2), suggesting a propensity to mature into cone photoreceptors. Retinal tissue sections collected from non-operated NHP controls were examined to confirm that the SC121, AMA and GFP staining was specific to the transplanted photoreceptor precursors; specific staining for these markers was not detected in the non-operated controls (Additional file 1: figure S2 E-H), or in operated eyes where primary antibodies were omitted (Additional file 1: figure S2 A-D).

## Discussion

This study demonstrates the safety and efficacy, in a naïve immunosuppressed non-human primate model, of sub-retinal transplantation of a cGMP-iPSC line previously demonstrated in immunodeficient murine host retina to generate a high percentage of photoreceptor precursors [15]. Upon sub-retinal transplantation of such cells, they displayed photoreceptor morphology and expressed specific markers of mature photoreceptors. Sub-retinal injection of these cells is well-tolerated, with no adverse effects on structure (OCT) or function (ERG) of the retina for 3 months following transplantation. Importantly, there was no evidence of an undesirable immune response, as shown by the lack of retinal oedema, inflammatory infiltrates or retinal/ choroidal lesions when examined by OCT and fundus imaging, the standard tools used to observe ocular inflammation in rodents [34], primates [35] and humans [36].

In addition, sub-retinal injection of photoreceptor precursors into NHP models with CoCl_2_-induced retinal degeneration was able to induce structural recovery and restored the EZ line on the OCT at 1 and 3 months post-transplantation. The EZ line corresponds to the mitochondria found within the ellipsoid layer of the outer portion of the photoreceptor inner segments [37], Recovery of the EZ (Fig. 4) suggests that sub-retinally injected retinal photoreceptor precursors successfully mediated structural recovery of the CoCl_2_ damaged photoreceptor layer. Although, a contribution from altered refractive and reflectance characteristics [38] cannot be excluded with certainty, restoration of the EZ was not present in controls up to 4 months following the procedure. This almost certainly suggests that transplanted photoreceptor precursors were able to mediate photoreceptor damage rescue. Unfortunately, correlation with histological results was technically difficult to achieve, as the region of interest was too small (1 mm^2^ in area) to be identified accurately on histology specimens.

Possible mechanisms by which photoreceptor recovery occurs include: (1) neuroprotective effects from secreted immune-modulatory factors, (2) maturation and integration of stem cell-derived photoreceptors to replace their damaged counterparts, and/ or (3) intracellular material exchange between transplanted cells and host photoreceptors [10, 39–41]. In this study there is evidence of photoreceptor structural recovery, possibly mediated by survival of photoreceptor cells, and the transplanted photoreceptors were able to survive in the NHP retina for up to 3 months in the presence of immunosuppression. This was demonstrated by the expression of GFP and two other human-specific cytoplasmic markers, SC121 and anti-mitochondrial antibody (AMA). Use of FAF enabled tracking of these GFP labelled photoreceptor precursors in the NHP eye and demonstrated that GFP fluorescent cells were detectable for up to 4-week post-transplantation by FAF and up to 3 months by IHC. In addition, these surviving photoreceptor precursors showed evidence of maturation into cone photoreceptors, with suggestion of integration into host ONL (as shown by the Hoechst 33342 negative gaps in the ONL that stained for human SC121/ AMA cytoplasmic staining). Confocal microscopy identified the presence of photoreceptors expressing both the human-specific markers and the cone-specific marker opsin M/L but did not identify cells co-expressing AMA and the rod specific marker rhodopsin. This is in contrast to previous work where cGMP-iPSC-derived photoreceptor precursors transplanted into immune-deficient murine models predominantly differentiated into rod photoreceptors [15]. In other NHP studies whereby retinal cell sheets and photoreceptor precursors were transplanted, the transplanted cells differentiated into cone photoreceptors first, with only one study reporting differentiation into rod photoreceptors only after an additional 28 days after the detection of cone photoreceptors [21, 50]. Thus, it is possible that with longer follow-up times post-transplantation, we might be able to observe transplanted cells differentiating into rod photoreceptors. It is probable also that this difference observed between NHP and murine transplants is due to the fundamental differences in retinal anatomy of the host retina. This difference in photoreceptor precursor differentiation will be investigated in future studies by staining with other rod markers, at longer post-transplantation time points.

Studies in NHP are of fundamental importance because assessment of biocompatibility in higher order animals that closely resemble human retinal physiology and structure is crucial for pre-clinical evaluation. Traditionally, rodent models have predominantly been used for pre-clinical evaluation of photoreceptor precursor transplants [42, 43]. However, rodents lack the cone-rich macula [44] found in humans, and as patients with macular degeneration will be one eventual target population for potential therapeutic intervention, the presence of a macula is desirable. Other large mammals such as cats [45] and pigs [46, 47] have been used for pre-clinical evaluation of retinal sheets, RPE monolayer and photoreceptor precursors but are also not ideal. Limitations of the cat model include the presence of the reflective tapetum lucidum, [48], while porcine eyes lack a fovea and are not optimal for pre-clinical studies due to inconsistencies in disease development patterns [48, 49]. In contrast, the ocular anatomy of NHPs resemble humans the most, making them most suitable for the evaluation of retinal cell-based therapies [44, 50–53]. To date there has been only one other study of photoreceptor precursor transplants in NHPs. CRX^+/tdTomato^ ESC-derived photoreceptor precursors [50] were transplanted sub-retinally into NHPs with laser ablated ONL. Similarly, they demonstrated cell survival in the sub-retinal space, with limited evidence of synaptic formation between photoreceptor precursors and host bipolar cells. Our present study is unique in that the photoreceptor precursors are derived from cGMP-grade iPSCs, which are cryo-preserved and shipped from USA. The cells are subsequently thawed in Singapore for further in vitro culture prior to transplantation. This proof-of-concept study therefore also demonstrates the feasibility of shipping cell products manufactured in one location to other countries, once approved, for clinical application.

One limitation of this study is the inability to co-stain the transplanted cells with human-specific nuclear markers such as human-specific nuclear antigen (HuNu) or human specific lamin B2 (hLMNB2) nuclear membrane protein to confirm transplanted cell survival and integration [15]. This difficulty arose from cross reactivity with the host NHP tissue (Additional file 1: figure S3). Thus, the possibility of retinal structural recovery due to indirect neurotrophic effects from the transplanted photoreceptor precursors or cytoplasmic material transfer or cell fusion cannot definitely be excluded [39–41]. To overcome this, alternative cell tracking methods such as CRISPR/Cas9 based genome detection will be explored [54, 55]. Future work also includes optimizing other human specific nuclear antibodies to differentiate host versus transplanted photoreceptor precursors, and the use of presynaptic markers to demonstrate better integration with the host retina [15, 56–58]. In addition, functional rescue should be examined by other modalities including multi-focal electro-retinography and visual evoked potentials. Another limitation is the inability to assess any immune privilege in the subretinal space, due to the need for immunosuppression for the xenograft transplantation. Future studies might include an allogenic cell resource with and without immunosuppression.

## Conclusions

This study firstly demonstrates the safety of subretinally transplantation of photoreceptor precursors derived from cGMP-grade iPSCs into healthy NHPs. When transplanted into a CoCl_2_-induced retinal degeneration model, the photoreceptor precursors could survive for up to 3 months post-transplant, mediating the structural recovery of the chemically damaged retina. Importantly, the transplanted photoreceptor precursors were able to mature and differentiate into cone photoreceptors, essential for the technique to have therapeutic potential in patients. This study provides proof of concept demonstrating the potential applicability of subretinal photoreceptor precursors suspension transplantation as a therapeutic strategy for IRDs.

## Supplementary Information

**Additional file 1**: **Table S1**: List of primary antibodies used in the study. **figure S1**: Estimation of GFP expression by FACS. After lentivirus infection, 95% of the RPC cells expressed GFP (left panel). There was no GFP in the non-infection control (right panel). **figure S2**: Controls for GFP, SC121 and AMA staining. (A-D) Secondary antibody only controls for Alexa 488 (B), Alexa 568 (C) and Alexa 647 (D). The background for all secondary antibodies appears to be high. (E-H) GFP, SC121 and anti-human mitochondrial antibody staining in non-operated NHP retina. None of the antibodies specifically stained anything in the non-operated control retina. The brightness/ contrast was artificially increased to identify any weakly stained cells. Hoechst 33342 was used to stain the INL and ONL layers (A, E). Scale bar 10 µm.** figure S3**: Cross reactivity of human lamin B2 (hLMNB2) antibody with the NHP retina. Tissue sections collected from NHP eyes post-photoreceptor precursor transplantation were stained with the human marker SC121 (B) and the human specific nuclear marker lamin B2 (C). The human nuclear marker antibody showed high cross-reactivity with the NHP tissue (C, D). Nuclei were stained with Hoechst 33,342 (H33342). Scale bar: 10 µm.

**Additional file 2**: **Video**: Surgical video of photoreceptor precursors subretinal injection under the NHP retina.

## Data Availability

All data generated or analysed during this study are included in this published article [and its supplementary information files].

## Abbreviations

IRDs: Inherited retinal diseases
RP: Retinitis pigmentosa
RPE: Retinal pigment epithelium
iPSC: Induced pluripotent stem cell
ESC: Embryonic stem cell
RPC: Retinal progenitor cell
cGMP: Current good manufacturing practice
OCT: Optical coherence tomography
NHP: Non-human primate
ONL: Outer nuclear layer
INL: Inner nuclear layer
PFA: Paraformaldehyde
BSA: Bovine serum albumin
FAF: Fundus autofluorescence
IR: Infrared
EZ: Ellipsoid zone
ERG: Electroretinography
AMA: Anti-mitochondrial antibody.
DA: Dark-adapted
LA: Light-adapted
