# Supplementary material for: cGMP-grade human iPSC-derived retinal photoreceptor precursor cells rescue cone photoreceptor damage in non-human primates
Source: Stem Cell Res Ther. 2021 Aug 19;12:464. doi: 10.1186/s13287-021-02539-8 (PMC8375124; doi:10.1186/s13287-021-02539-8)

Additional table 1: List of primary antibodies used in the study.

Additional Table 1: List of primary antibodies used in this study

Target gene	Vendor and Cat#	Species	Dilution	Antigen retrieval
LHX2	Santa Cruz, sc-5172431	Mouse	1:20	NA
Recoverin (RCVN)	Millipore, AB5585	Rabbit	1:500	NA
CRX	Genetex, GTX124188	Rabbit	1:200	NA
OTX2	Abcam, ab92326	Rabbit	1:200	NA
NRL	R&D System, BAF2945	Goat	1:200	NA
Cone Arrestin	EMD Millipore, AB15282	Rabbit	1:100	NA
Rhodopsin (RHO)	Millipore, MAB5356	Mouse	1:500	NA
GFP	Abcam, ab290	Rabbit	1:500	Citrate buffer pH 7.0
STEM121 (SC121)	Takara Bioscience, Y40410	Mouse	1:500	Citrate buffer pH 7.0
Anti- mitochondrial antibody clone 113-1	Merck Millipore, MAB1273	Mouse	1:100	Citrate buffer pH 7.0
Rhodopsin	Abcam, ab221664	Rabbit	1:500	Tris- EDTA buffer pH 9.0
Opsin MW/MW2/LW (K-19)	Santa Cruz Biotechnology, sc-2117	Goat	1:100	Citrate buffer pH 7.0

Additional figure 1: Estimation of GFP expression by FACS. After lentivirus infection, 95% of the RPC cells expressed GFP (left panel). There was no GFP in the non- infection control (right panel).

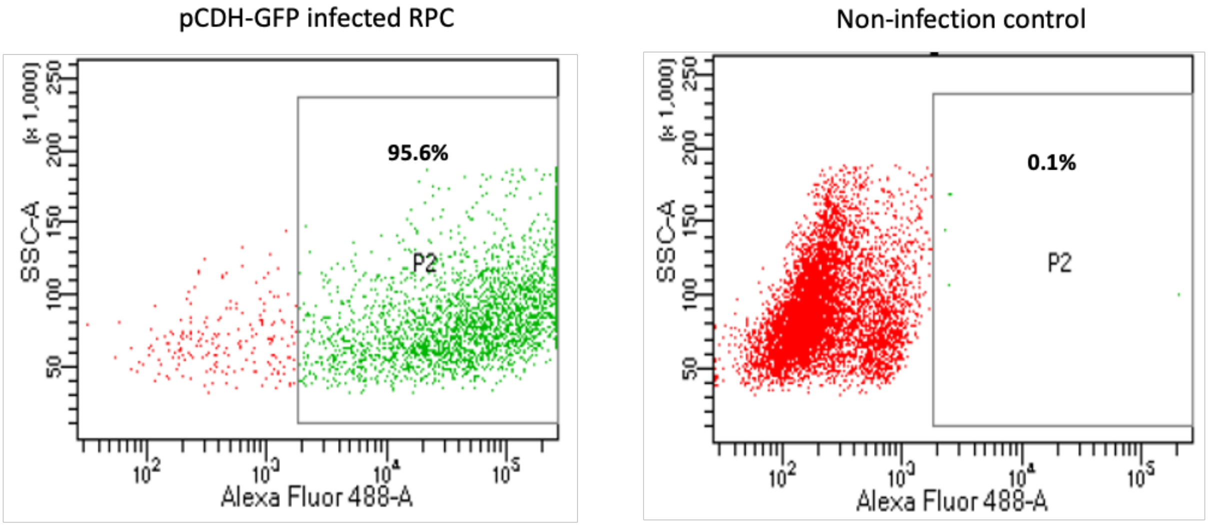

Additional figure 2: Controls for GFP, SC121 and AMA staining. (A-D) Secondary antibody only controls for Alexa 488 (B), Alexa 568 (C) and Alexa 647 (D). The background for all secondary antibodies appears to be high. (E-H) GFP, SC121 and anti- human mitochondrial antibody staining in non- operated NHP retina. None of the antibodies appear to specifically stain anything in the non- operated control retina. The brightness/ contrast was artificially increased to identify any weakly stained cells. Hoechst 33342 was used to stain the INL and ONL layers (A, E). Scale bar 10 μm.

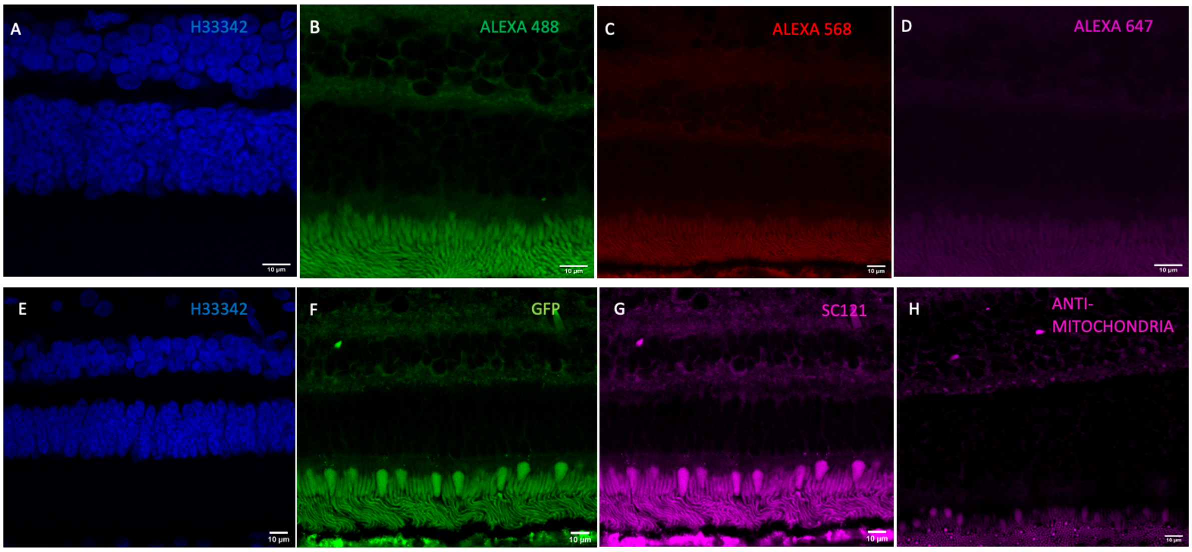

Additional figure 3: Cross reactivity of human lamin B2 (hLMNB2) antibody with the NHP retina. Tissue sections collected from NHP eyes post photoreceptor precursor transplantation were stained with the human marker SC121 (B) and the human specific nuclear marker lamin B2 (C). The human nuclear marker antibody showed high cross- reactivity with the NHP tissue (C, D). Nuclei were stained with Hoechst 33342 (H33342). Scale bar: 10 μm.

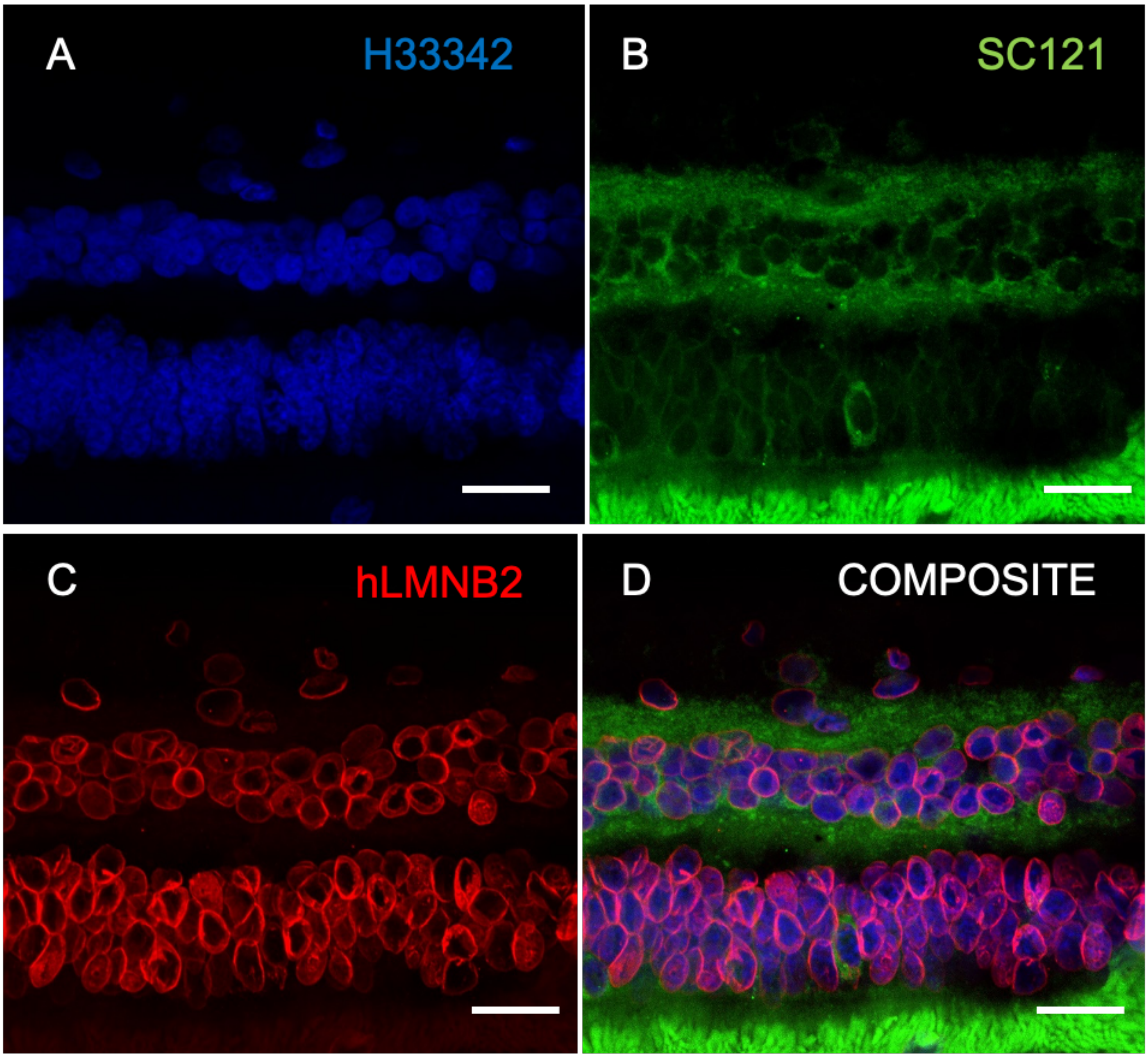

Supplement: Supplementary file 1 — Additional file 1: Table S1: List of primary antibodies used in the study. figure S1: Estimation of GFP expression by FACS. After lentivirus infection, 95% of the RPC cells expressed GFP (left panel). There was no GFP in the non-infection control (right panel). figure S2: Controls for GFP, SC121 and AMA staining. (A-D) Secondary antibody only controls for Alexa 488 (B), Alexa 568 (C) and Alexa 647 (D). The background for all secondary antibodies appears to be high. (E-H) GFP, SC121 and anti-human mitochondrial antibody staining in non-operated NHP retina. None of the antibodies specifically stained anything in the non-operated control retina. The brightness/ contrast was artificially increased to identify any weakly stained cells. Hoechst 33342 was used to stain the INL and ONL layers (A, E). Scale bar 10 µm. figure S3: Cross reactivity of human lamin B2 (hLMNB2) antibody with the NHP retina. Tissue sections collected from NHP eyes post-photoreceptor precursor transplantation were stained with the human marker SC121 (B) and the human specific nuclear marker lamin B2 (C). The human nuclear marker antibody showed high cross-reactivity with the NHP tissue (C, D). Nuclei were stained with Hoechst 33,342 (H33342). Scale bar: 10 µm. [file 13287_2021_2539_MOESM1_ESM.pdf]
